# Editorial: Myelodysplastic neoplasm and acute myeloid leukemia: multi-omics approaches and precision medicine

**DOI:** 10.3389/fonc.2026.1860744

**Published:** 2026-05-15

**Authors:** Teresa De Souza Fernandez, Chung Hoow Kok

**Affiliations:** 1 Cytogenetic Laboratory, Cell and Gene Therapy Program, Instituto Nacional de Câncer, Rio de Janeiro, Brazil; 2 Centre for Cancer Biology, College of Health, School of Medicine, Adelaide University, Adelaide, SA, Australia; 3 Data and Bioinformatics Innovation, Department of Genetics and Molecular Pathology, SA Pathology, Adelaide, SA, Australia

**Keywords:** acute myeloid leukemia, diagnosis, multi-omics, myelodysplastic neoplasms, precision medicine, prognosis

Myelodysplastic neoplasms (MDS) and acute myeloid leukemia (AML) are heterogeneous myeloid malignancies characterized by chromosomal abnormalities, genetic variants, epigenetic alterations, and clinical variability (1, 2). Recent advances in multi-omics technologies including cytogenomics, genomics, epigenomics, transcriptomics, proteomics, metabolomics and single cell omics approaches have substantially expanded our understanding of cancer biology. In parallel, the integration of molecular, cytogenetic, bioinformatic, and clinicopathologic data is reshaping diagnosis, and therapeutic stratification (3, 4). In MDS, allogeneic hematopoietic cell transplantation remains the only potentially curative treatment for eligible patients, underscoring the importance of better biomarkers and more precise risk assessment (5, 6).

This Research Topic brings together 10 articles that collectively highlight the expanding role of multi-omics and integrative diagnostic strategies in advancing our understanding of MDS and AML. Taken together, these articles highlight the growing clinical relevance of high-resolution genomic profiling, molecularly informed disease classification, immune and microenvironmental biology, artificial intelligence-assisted diagnosis, and emerging biomarkers with translational potential.

A major theme across the Research Topic is the increasing diagnostic value of integrated cytogenomic and molecular analysis in myeloid neoplasms. Cytogenetics plays an important role in myeloid neoplasms being incorporated in the risk prognostic scales, and for the genes identification involved in the leukemogenesis (1, 2, 7–9). Tsai et al. showed that the optical genome mapping (OGM) can complement conventional G-banding by improving the characterization of structural variants in leukemia. This study highlights the practical value of higher-resolution genomic technologies for resolving chromosomal complexity that may be incompletely defined by standard cytogenetics alone. Similarly, Mikkilineni et al. reported two new myeloid neoplasm cases with *MECOM::ETV6*. Using a comprehensive approach including: histopathological, G-banding, FISH, NGS and it was also performed a review of literature for the structural abnormalities resulting in gene fusion between *MECOM* locus in hematologic neoplasms in public data bases. This study highlights the value of integrating histopathology, conventional cytogenetics, FISH, and NGS to define rare clinically relevant rearrangements. Chang et al. further expanded the spectrum of recurrently detectable abnormalities by reporting a novel translocation t (5,17)(q24;q11.2) involving *NF1::SCAMP5* identified by RNA-seq and confirmed by Sanger sequencing. In another case-based contribution, Wang and Jin showed for the first time a MDS/MPN patient harboring *SF3B1* mutation coexisting with the mutations in *ASXL1*, *JAK2* and *CBL*. This case highlights the importance of integrative molecular-morphological framework that allowed a precision diagnosis evaluation.

Beyond diagnosis, the Research Topic also addresses mechanisms of disease evolution. Shan et al. investigated key potential factors associated with disease progression in Aplastic Anemia (AA), MDS, and AML. These three diseases have complex pathogenic mechanisms and share clinical and molecular features. Identifying the factors that associated with disease progression is essential for preventing disease evolution to an advanced stage, and therefore critical for reducing treatment challenges (high relapse rates, drug resistance) and for improvement of therapeutic outcomes. The authors showed that *POLG* and *MAP2K7* play a significant role throughout the progression from AA to MDS and to AML through the modulation of immune regulatory pathways, influencing disease transformation and progression. It is important to note that patients who have AML secondary from MDS have in general an unfavorable clinical evolution (10, 11). This critical clinical outcome is also observed in patients with *TP53* mutated in MDS and AML, that remains a major clinical challenge and is often associated with adverse outcomes (12, 13). Complementing this theme, Albakri conducted a review that explored the mechanisms of immune dysfunction in *TP53*-mutated MDS and AML, focusing on the interplay between genetic lesions, tumor microenvironment, metabolism, and emerging therapeutic strategies. This review underscores the need for combinatorial approaches that integrate targeted, immunomodulatory, and metabolism-directed therapy as a transformative approach to improve patient outcomes in this biologically high-risk disease.

This Research Topic also highlights how precision medicine is being extended beyond sequencing-based approaches. In acute promyelocytic leukemia (APL), rapid diagnosis is essential because identification of *PML::RARA* has immediate therapeutic implications, particularly for the use of all-trans retinoic acid (ATRA) (14). Yan et al. showed the importance of accurately diagnosing APL which accounts for 10-15% of all AML cases by developing an innovative artificial intelligence-based image analysis approach capable of identifying APL-associated fusion patterns directly from pathological images. Both classical APL and its variants differ in their biological characteristics and their response to ATRA. This study highlights how computational pathology may augment molecular diagnostics and accelerate clinically actionable classification.

Additional contributions broaden the translational scope of this topic. Huang and Pang reported successful remission induction with venetoclax and azacitidine in an elderly patient with early T-cell precursor/myeloid mixed-phenotype acute leukemia (MPAL), a rare subtype in myeloid neoplasms for which evidence-based treatment guidance remains limited. The search for novel biomarkers and therapeutic targets in MDS and AML continues to drive research in the field. In this context, Gao et al. used Circle-seq and RNA-seq to characterize extrachromosomal circular DNA (eccDNA) in patients with AML and healthy controls. Their study provides an important overview of the eccDNA landscape in AML and suggests that eccDNA may serve as both a biomarker and a therapeutic target. Complementing this, molecular profiling studies provide important insights into population-specific mutational patterns, which may vary geographically and have implications for diagnosis and treatment. Consistent with this, Liao et al. used targeted next-generation sequencing to characterize distinct mutational landscapes in Chinese patients with AML, MDS, and CMML, underscoring the importance of population-specific molecular profiling for improving diagnosis, refining risk assessment, and informing precision medicine strategies.

Overall, this Research Topic demonstrates how the field is moving from single-platform testing toward integrated, multi-omics models in myeloid neoplasms. Collectively, the studies in this Research Topic reinforce the concept that precision medicine in MDS and AML depends not only on the identification of genetic variants, but also on the interpretation of structural variation, transcriptional consequences, immune context, clinicopathologic phenotype, and, increasingly, image-derived computational data. Together, these contributions open new avenues for furthers research aimed at improving diagnosis, refining disease classification, guiding the development of individualized treatment strategies for both pediatric and adult patients with MDS and AML, marking the dawn of a new era in precision medicine.
